# 

**Published:** 1900-11

**Authors:** 


### BOOKS RECEIVED.

A Manual of Otology. By Gorham Bacon, A. M., M. D., Professor of Otology in Cornell University Medical College, New York. With an introductory chapter by Clarence J. Blake, M. D., Professor of Otology in the Harvard Medical School, Boston. In one handsome 12mo. volume of 422 pages, with 114 engravings and three colored plates. Lea Brothers & Co., Publishers, Philadelphia and New York. 1900. Price, cloth, $2.25, net.

Dunglison's Dictionary of Medical Science. Containing a full explanation of the various subjects and terms of Anatomy, Physiology, Medical Chemistry, Pharmacy, Pharmacology, Therapeutics, Medicine, Hygiene, Dietetics, Pathology, Surgery, Ophthalmology, Otology, Laryngology, Dermatology, Gynecology, Obstetrics, Pediatrics, Medical Jurisprudence, Dentistry, Veterinary Science, etc., etc. By Robley Dunglison, M. D , LL. D., late Professor of the Institutes of Medicine in the Jefferson Medical College of Philadelphia Edited by Richard J. Dunglison, A. M., M. D. New (22d) edition, thoroughly revised, greatly enlarged and improved, with the pronunciation, accentuation and derivation of the terms. In one magnificent imperial octavo volume of 1350 pages. Lea Brothers & Co., Publishers, Philadelphia and New York. 1900. Cloth, $7.00, net; full leather, $8.00 net.

Rhinology, Laryngology and Otology, and their Significance in General Medicine. By E. P. Friedrich, M. D., Privadocent at the University of Leipzig. Authorised translation from the German. Edited by H. Holbrook Curtis, M. D., Consulting Surgeon to the New York Nose and Throat Hospital and to the Diphtheria and Scarlet Fever Hospitals. Octavo, pp. 348. Philadelphia and London. W. B. Saunders & Co. 1900. Price $2.50 net.

Bacteriology and Surgical Technique for Nurses. By Emily M. A. Stoney, Superintendent of the Training School for Nurses, St. Anthony's Hospital, Rock Island, Ill., etc. Duodecimo, pp. 190. Illustrated. Philadelphia: W. B. Saunders & Co. 1900. Price, $1.25, net.

Medical and Surgical Nursing. A treatise on modern nursing from the physician's and surgeon's standpoint, for the guidance of graduate and student nurses, together with practical instruction in the art of cooking for the sick. Edited by H. J. O'Brien, M. D., Professor of Clinical Surgery, Hamline University; Surgeon to St. Joseph's Hospital, St. Paul, etc. 12mo. pp. ix.—287. New York: G. P. Putnam's Sons. 1900 [Price, $1.50.]

Deaver's Surgical Anatomy. A Treatise on Human Anatomy in its Application to the Practice of Medicine and Surgery. By John B. Deaver, M. D., Surgeon-in-Chief to the German Hospital, Philadelphia. In three royal 8vo volumes of more than 600 pages each, containing about 450 full-page plates, nearly all drawn for this work from original dissections. Volume I.—Upper extremity; back of neck, shoulder, and trunk; cranium; scalp; face. Volume II.—Neck; mouth, pharynx, larynx, nose; orbit; eyeball; organ of hearing; brain; female perineum; male perineum. Philadelphia: P. Blakiston's Son & Co., 1012 Walnut street. 1900. Price, handsome cloth, $21.00; full sheep, $24.00; half green Morocco, marbled edges, $24.00; half Russia, gilt, marbled edges, $27.00 net.

A Reference Handbook of the Medical Sciences. Embracing the Entire Range of Scientific and Practical Medicine and Allied Science. By various writers. A new edition, completely revised and rewritten. Edited by Albert H. Buck, M D., New York City. Volume I.—AAC–BLA. Illustrated by numerous chromo-lithographs and 498 fine half-tone and wood engravings. Nine volumes, imperial 8vo. New York: William Wood & Co. 1900. Price, muslin, $6.00 per volume; leather, $7.00 per volume; half Morocco, $8.00 per volume.

A Treatise on Fractures and Dislocations. For Practitioners and Students. By Lewis A. Stimson, B A., M. D., Professor of Surgery in Cornell University Medical College, New York. New (3d) edition. In one 8vo volume of 842 pages, with 336 engravings and 32 full-page plates. Philadelphia and New York: Lea Brothers & Co. 1900. Price, cloth, $5.00 net; leather, $6.00 net.

A Text-Book of the Practice of Medicine. By James M. Anders, M. D., Ph D., LL. D., Professor of the Practice of Medicine and of Clinical Medicine in the Medico-Chirurgical College, Philadelphia; Attending Physician to the Medico-Chirurgical and Samaritan Hospitals, Philadelphia. Octavo, pp. 1263. Illustrated. Fourth edition, thoroughly revised. Philadelphia and London: W. B. Saunders & Co. 1900. Price, cloth, $5.00 net; sheep and half Morocco, $6.50.

Modern Surgery. General and Operative. By John Chalmers DaCosta, M. D., Professor of the Principles of Surgery and of Clinical Surgery, Jefferson Medical College, Philadelphia; Surgeon to the Philadelphia Hospital and to St. Joseph's Hospital, Philadelphia. Octavo, pp. 1117. With 493 illustrations. Third edition, revised and enlarged. Philadelphia and London: W. B. Saunders & Co. 1900. Price, cloth, $5.50 net; half Morocco, $6.00.

Modern Medicine. By Julius L. Salinger, M. D., Demonstrator of Clinical Medicine, Jefferson Medical College; Hematologist to the Jefferson Medical College Hospital; Pathologist to the Lying-in Charity Hospital, Philadelphia; Assistant Pathologist to the Philadelphia Hospital. Octavo, pp. 801. Illustrated. Philadelphia and London: W. B. Saunders & Co. 1900. Price, cloth, $4.00 net.

Twentieth Century Practice. An International Encyclopedia of Modern Medical Science. By Leading Authorities of Europe and America. Edited by Thomas L. Stedman, M. D., New York City. In twenty volumes. Volume XX. "Tuberculosis, Yellow Fever and Miscellaneous. General Index." New York: William Wood & Company. 1900.

Studies in the Psychology of Sex. The Evolution of Modesty.—The Phenomena of Sexual Periodicity.—Auto-Erotism. By Havelock Ellis. Pages xii.—275. Sold only to physicians and lawyers. Philadelphia: F. A. Davis Company. 1900. Cloth, $2.00 net.

A Text-Book of the Diseases of Women. By Henry J. Garrigues, A. M., M. D., Gynecologist to St Mark's Hospital, New York City. Octavo volume of 756 pages, with 367 illustrations. Third edition, thoroughly revised. Philadelphia and London: W. B. Saunders & Co. 1900. Price, cloth, $4.00 net; sheep or half Morocco, $5.50 net.

An American Text-Book of Physiology. By ten of the Leading Physiologists of America. Edited by William H. Howell, Ph. D., M. D., Professor of Physiology in the Johns Hopkins University, Baltimore. Octavo, 1052 pages, illustrated. Second edition, revised. Volume I. Philadelphia: W. B. Saunders & Co. 1900. Price, cloth, $6.00 net; sheep or half Morocco, $7.00 net.

Diet Lists and Sick-Room Dietary. By Jerome B. Thomas, M. D., Visiting Physician to the Home for Friendless Women and Children and to the Newsboys' Home; Assistant Bacteriologist, Brooklyn Health Department. Philadelphia: W. B. Saunders & Co. 1900. Price, cloth, $1.50.

Taylor on Genito-Urinary and Venereal Diseases and Syphilis. The Pathology and Treatment of Genito-Urinary and Venereal Diseases and Syphilis. By Robert W. Taylor, A. M., M. D., Clinical Professor of Venereal Diseases in the College of Physicians and Surgeons, New York. New (2d) edition. Octavo volume of 720 pages, with 135 engravings and 27 full-page plates in colors and monotone. Philadelphia and New York: Lea Brothers & Co. 1900. Cloth, $5.00 net; leather, $6.00

Heart Disease in Childhood and Youth. By Charles W. Chapman, M. D., Durh., M. R. C. P. Lond., Physician to the National Hospital for Diseases of the Heart, Soho Square, W , etc. With an introduction by Sir Samuel Wilks, Bart., M. D., F. R. S., Physician Extraordinary to H. M. the Queen, etc. Duodecimo, pp. 101. London: Medical Publishing Co. 1900. Price, 3s. 6.

Physical Diagnosis of Diseases of the Chest. By Richard C. Cabot, M. D., Physician to Out-patients, Massachusetts General Hospital; Assistant in Clinical Medicine, Harvard Medical School. Octavo, pp. xv.—310. 142 illustrations. New York: William Wood & Co. 1900.

Les Maladies Qu'on Soigne A. Berck. Abcès froids, adénites, ostéites, Tumeurs Blanches, Coxalgie, Mal de Pott, Scoliose, Luxation Congénitale de la Hanche, Pied Bot, etc. Par F. Calot, Chirurgien en chef de l'Hopital Rothschild, etc., etc. Pages, 433. Paris: G. Masson, Editeur, Librairie de L'Academie de Médecine, 120 Boulevard Saint Germain. 1900.

A Text-Book Upon the Pathogenic Bacteria. For Students of Medicine and Physicians. By Joseph McFarland, M. D., Professor of Pathology in the Medico-Chirurgical College, Philadelphia. Octavo, 621 pages, illustrated. Third edition, revised and enlarged. Philadelphia and London: W. B. Saunders & Co. 1900. Price, cloth, $3.25 net.

Pathology and Morbid Anatomy. By T. Henry Green, M. D., F. R. C. P., Physician and Special Lecturer on Clinical Medicine at Charing Cross Hospital, etc. New (9th) American from ninth English edition. Revised and enlarged by H. Montague Murray, M. D., F. R. C. P., Lecturer on Pathology and Morbid Anatomy at Charing Cross Hospital. Revised for America by Walton Martin, Ph. B., M. D., of the College of Physicians and Surgeons, New York City. Octavo volume of 578 pages, with 4 colored plates and 339 engravings. Philadelphia and New York: Lea Brothers & Co. Price, cloth, $3.25 net

The American Illustrated Medical Dictionary. A New and Complete Dictionary of the Terms Used in Medicine, Surgery, Dentistry, Pharmacy, Chemistry and the Kindred Branches. With their Pronunciation, Derivation, and Definition. Including much Collateral Information of an Encyclopedic Character. By W. A. Newman Dorland, A. M., M. D., Assistant Obstetrician to the University of Pennsylvania Hospital; Editor of the American Pocket Medical Dictionary. With numerous illustrations and 24 colored plates. Philadelphia and London: W. B. Saunders & Co. 1900. Price, $4.50, plain; $5.00, index.

A Text-Book of Pathology. By Alfred Stengel, M. D., Professor of Clinical Medicine in the University of Pennsylvania; Physician to the Philadelphia Hospital; Physician to the Children's Hospital, Philadelphia, etc. Octavo, pp. 873. With 372 illustrations. Third edition, revised. Philadelphia and London: W. B. Saunders & Co. 1900. Price, cloth, $5.00 net; half-Morocco, $6.00 net.

Saunders's Question Compends. Essentials of Histology. By Louis Leroy, B. S., M. D., Professor of Histology and Pathology, in Vanderbilt University, Medical and Dental Departments; City Bacteriologist to Nashville, Tenn.; Bacteriologist to the State of Tennessee. Arranged with questions following each chapter. Duodecimo, pp. 231, 72 illustrations. Philadelphia: W. B. Saunders & Co. 1900. Price, $1.00 net.

Saunders's Pocket Medical Formulary. With an appendix. By William M. Powell, M. D., anthor of "Essentials of Diseases of Children." Sixth edition, thoroughly revised. Philadelphia: W. B. Saunders & Co. 1900. Price, $2.00, net.

A Pocket Text-Book of Diseases of the Eye, Ear, Nose and Throat, for students and practitioners. By William L. Ballinger, M. D., Assistant Professor of Otology, Rhinology and Laryngology in the College of Physicians and Surgeons, Chicago, etc., and A. G. Wippern, M. D., Professor of Ophthalmology and Otology in the Chicago Eye, Ear, Nose and Throat College. In one handsome 12mo volume of 525 pages, with 150 engravings and 6 full-page colored plates. Philadelphia and New York: Lea Brothers & Co., Publishers. 1900. Price, cloth, $2.00, net; flexible red leather, $2.50, net.

